# High-resolution characterization of the temporal and spatial distribution of antimicrobial resistance in *Escherichia coli* from pigs

**DOI:** 10.1093/jac/dkag196

**Published:** 2026-06-19

**Authors:** Sam Abraham, Zheng Zhou Lee, David Jordan, Marc Stegger, André Becker S Saidenberg, Kittitat Lugsomya, Terence Lee, Shewli Mukerji, Lance Price, Kim Nairn, Rebecca J Abraham

**Affiliations:** Centre for Biosecurity and One Health, Harry Butler Institute, Murdoch University, 90 South Street, Murdoch, Western Australia 6150, Australia; Antimicrobial Resistance and Infectious Diseases Laboratory, School of Medical, Molecular and Forensic Sciences, Murdoch University, 90 South Street, Murdoch, Western Australia 6150, Australia; Centre for Biosecurity and One Health, Harry Butler Institute, Murdoch University, 90 South Street, Murdoch, Western Australia 6150, Australia; Antimicrobial Resistance and Infectious Diseases Laboratory, School of Medical, Molecular and Forensic Sciences, Murdoch University, 90 South Street, Murdoch, Western Australia 6150, Australia; Centre for Biosecurity and One Health, Harry Butler Institute, Murdoch University, 90 South Street, Murdoch, Western Australia 6150, Australia; Antimicrobial Resistance and Infectious Diseases Laboratory, School of Medical, Molecular and Forensic Sciences, Murdoch University, 90 South Street, Murdoch, Western Australia 6150, Australia; Centre for Biosecurity and One Health, Harry Butler Institute, Murdoch University, 90 South Street, Murdoch, Western Australia 6150, Australia; Antimicrobial Resistance and Infectious Diseases Laboratory, School of Medical, Molecular and Forensic Sciences, Murdoch University, 90 South Street, Murdoch, Western Australia 6150, Australia; Department of Sequencing and Bioinformatics, Statens Serum Institut, 5 Artillerivej, Copenhagen S DK-2300, Denmark; Department of Sequencing and Bioinformatics, Statens Serum Institut, 5 Artillerivej, Copenhagen S DK-2300, Denmark; Antimicrobial Resistance and Infectious Diseases Laboratory, School of Medical, Molecular and Forensic Sciences, Murdoch University, 90 South Street, Murdoch, Western Australia 6150, Australia; Faculty of Veterinary Medicine, Mahanakorn University of Technology, Bangkok 10530, Thailand; Centre for Biosecurity and One Health, Harry Butler Institute, Murdoch University, 90 South Street, Murdoch, Western Australia 6150, Australia; Antimicrobial Resistance and Infectious Diseases Laboratory, School of Medical, Molecular and Forensic Sciences, Murdoch University, 90 South Street, Murdoch, Western Australia 6150, Australia; Centre for Biosecurity and One Health, Harry Butler Institute, Murdoch University, 90 South Street, Murdoch, Western Australia 6150, Australia; Antimicrobial Resistance and Infectious Diseases Laboratory, School of Medical, Molecular and Forensic Sciences, Murdoch University, 90 South Street, Murdoch, Western Australia 6150, Australia; Department of Sequencing and Bioinformatics, Statens Serum Institut, 5 Artillerivej, Copenhagen S DK-2300, Denmark; Portec Veterinary Services, 1/8 Tomlinson Rd, Welshpool, Western Australia 6106, Australia; Centre for Biosecurity and One Health, Harry Butler Institute, Murdoch University, 90 South Street, Murdoch, Western Australia 6150, Australia; Antimicrobial Resistance and Infectious Diseases Laboratory, School of Medical, Molecular and Forensic Sciences, Murdoch University, 90 South Street, Murdoch, Western Australia 6150, Australia

## Abstract

**Objectives:**

Livestock are recognized reservoirs of antimicrobial resistance (AMR). However, current surveillance often overlooks key ecological aspects such as spatial–temporal patterns and quantification of shedding levels of resistant bacteria. In particular, early detection of low-level shedding of resistance to critically important antimicrobials (CIAs), including extended-spectrum cephalosporins (ESCs) and fluoroquinolones (FQs), remains limited. Using commensal *Escherichia coli* as an indicator, we applied a high-throughput Robotic Antimicrobial Susceptibility Platform (RASP) to assess phenotypic resistance in isolates from 900 samples collected across 10 pig herds over 3 years.

**Methods:**

Quantitative assessments of antimicrobial resistance (cfu/g) were performed using selective agars containing antimicrobials, with plating and data capture (colony counting) automated on the RASP. Broth microdilution and whole-genome sequencing were performed on CIA-R *E. coli* using RASP.

**Results:**

Persistent resistance to ampicillin and tetracycline (∼5.8 log_10_ cfu/g) showed minimal variation between herds and years. Gentamicin resistance declined significantly (−0.23 log_10_ cfu/g/year, *P* < 0.0001), while ESC resistance rose significantly (0.16 log_10_ cfu/g/year, *P* = 0.015), although some herds showed no ESC shedding. Ciprofloxacin resistance was detected in 58% of samples but generally at lower levels (∼2.1 log_10_ cfu/g) with herd-level variability. Genomic analysis identified FQ-resistant sequence types ST744 and ST167 with global phylogenetic links, and ESC resistance was associated with *bla*_CTX-M-1_ on IncI1 plasmids.

**Conclusions:**

These findings reveal the ecological complexity of AMR in livestock and highlight limitations of standard surveillance in detecting rare resistances. Our study demonstrates how high-throughput robotics integrated with robust field design can enhance AMR monitoring and inform One Health strategies for mitigation.

## Introduction

Antimicrobial resistance (AMR) is a major global health challenge, contributing to an estimated five million deaths annually.^1^ Resistance to critically important antimicrobials (CIAs), including carbapenems, extended-spectrum cephalosporins (ESCs) and fluoroquinolones (FQs), threatens the effectiveness of last-resort treatments for severe bacterial infections.^2^ Within the One Health continuum, livestock production systems significantly contribute to the emergence and spread of AMR through antimicrobial use that selects for resistant bacteria transmissible to humans via direct contact, environmental pathways and the food chain.^3^ Commensal *Escherichia coli* is therefore a key sentinel organism for resistance surveillance due to its ubiquity and role as a reservoir of resistance genes.^3^ In this study, ESC resistance is used as a phenotypic classification and includes *E. coli* isolates producing extended-spectrum β-lactamases (ESBLs) and/or plasmid-mediated AmpC β-lactamases, where relevant. Accordingly, the term ‘ESC-resistant *E. coli*’ is used throughout unless specific resistance mechanisms are explicitly described.

Traditional AMR surveillance methods have limitations, including small sample sizes and reliance on a single isolate per sample, which fail to capture the extensive variability of resistance within and between animals and populations.^4^ This is particularly problematic for low-prevalence resistance traits, such as those associated with CIAs. These limitations hinder early detection and the ability to track emerging resistance trends at a granular level. To address these challenges, advanced, integrated phenotypic–genomic methodologies are necessary.

High-resolution robotic platforms offer a transformative approach to AMR surveillance. By automating key processes, these platforms can analyse large numbers of samples with unprecedented efficiency and accuracy. They enable the quantification of resistant phenotypes at the population level and facilitate detailed genomic (characterization of isolates, providing insights into resistance mechanisms, genetic diversity and potential transmission pathways). Such methodologies are critical for improving the precision of surveillance efforts and enhancing their utility for public health decision-making.^5,6^

Australia provides a unique context for studying AMR in livestock. The country’s stringent regulations on antimicrobial use in agriculture, including the prohibition of FQs and carbapenems in food-producing animals, have resulted in relatively low levels of resistance compared to other regions.^7^ However, the emergence of resistance to CIAs, such as ESCs, in livestock remains a concern, particularly given the potential for global transmission of resistance genes through wildlife, humans and the environment. Monitoring these trends is essential for safeguarding public health and ensuring the continued efficacy of CIAs.

This study leverages a high-throughput platform to investigate AMR in *E. coli* from Australian pig herds over a 3-year period. By integrating phenotypic resistance testing, quantitative colony enumeration and whole-genome sequencing (WGS), the study provides a comprehensive assessment of resistance trends at the herd level. Specific objectives include quantifying temporal changes in resistance to CIAs, characterizing the genetic basis of resistance in *E. coli* isolates and evaluating their phylogenetic relationships to global strains.

The findings aim to address critical knowledge gaps in AMR dynamics within livestock populations, offering actionable insights for antimicrobial stewardship. By demonstrating the utility of high-resolution surveillance tools, this research contributes to the broader goal of mitigating AMR risks through evidence-based policies and practices within the One Health framework.

## Materials and methods

**Figure 1.**
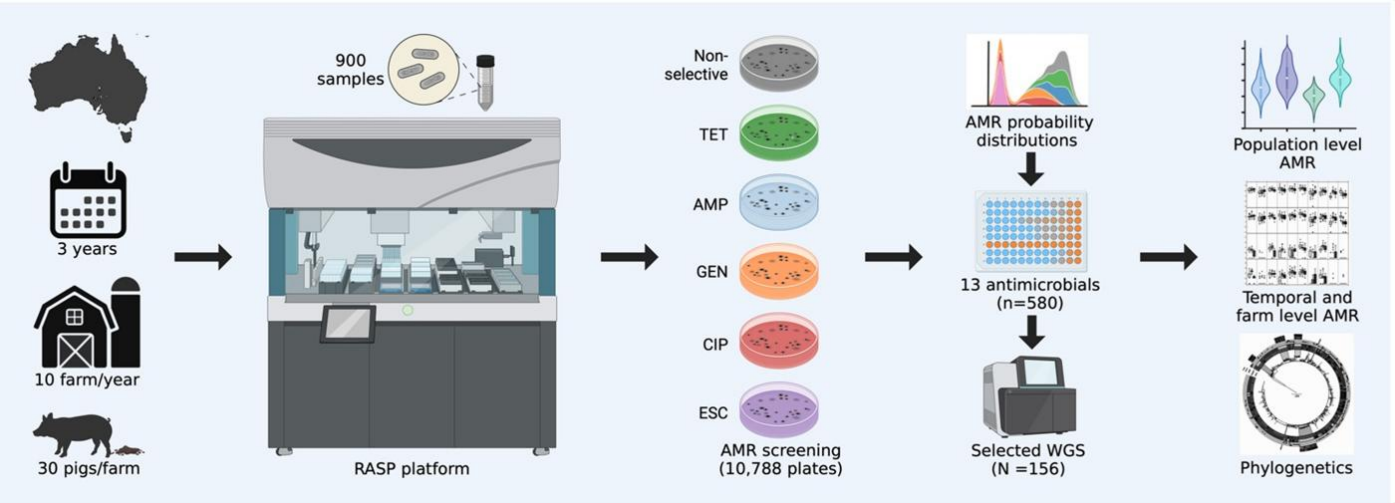
Schematic overview of the 3-year Australian pig study. Overview of sampling strategy across farms and individual pigs, highlighting the high-throughput automation and AMR screening of isolates. Post-screening, subsets of isolates were screened for MICs and genome sequenced. Abbreviations: Robotic Antimicrobial Susceptibility Platform (RASP), ampicillin (AMP), tetracycline (TET), gentamicin (GEN), ciprofloxacin (CIP) and extended-spectrum cephalosporin (ESC).

A schematic overview of the study and the schematic diagram illustrating the RASP design and workflow are shown in Figure 1.

### Sample collection and processing

Faecal samples were collected annually from 10 commercial pig herds in Australia over 3 years (2019–2021), with farms and veterinarians agreeing to participate in the study for the full duration. Each herd contributed 30 samples per year (one herd provided 29 samples in 2019), yielding a total of 899 samples. Samples were collected under veterinary supervision from multiple pens using sterile tools, transported on ice and processed within 16 h. In the laboratory, 2 g of faeces was homogenized in 18 mL phosphate-buffered saline, followed by 10-fold serial dilutions to obtain quantifiable colony counts.

### Quantification of AMR

The Robotic Antimicrobial Susceptibility Platform (RASP) was used to automate plating, incubation, imaging and enumeration of *E. coli* resistant to selected antimicrobials, improving throughput and reproducibility.^5,6^ Faecal suspensions were plated on CHROMagar ECC for selective isolation of *E. coli*, with species confirmation by MALDI-TOF MS and on CHROMagar ECC supplemented with ampicillin (32 mg/L), tetracycline (16 mg/L), gentamicin (16 mg/L) or ciprofloxacin (4 mg/L) at CLSI breakpoint concentrations; ESC resistance was assessed using CHROMagar ESBL according to a previously validated protocol.^8^ Plates were incubated aerobically at 37°C for 16–20 h, imaged using RASP-integrated cameras, and colonies were quantified by image analysis, with resistance levels expressed as cfu/g after accounting for dilution factors.

### Phenotypic characterization

A subset of colonies exhibiting resistance on ciprofloxacin- and ESC-infused agars was selected for phenotypic confirmation. Single colonies were isolated and subjected to broth microdilution testing using the RASP protocols according to CLSI guidelines.^6,9^ This method determined minimum inhibitory concentrations (MICs) for a panel of 13 antimicrobials (ampicillin, apramycin, cefoxitin, ceftiofur, ceftriaxone, chloramphenicol, ciprofloxacin, colistin, florfenicol, gentamicin, streptomycin, tetracycline, sulfamethoxazole/trimethoprim), spanning 7 classes. The panel included aminoglycosides, β-lactams, folate pathway inhibitors, FQs, phenicols, polymyxins and tetracyclines.

MICs were interpreted according to CLSI, European Committee on Antimicrobial Susceptibility Testing (EUCAST v12.0) and NARMS breakpoints.^9–11^  *E. coli* ATCC 25922 was used as the reference strain for the MIC method and breakpoint interpretation.

### Genomic analyses

WGS (Illumina) was performed on 151 isolates representing diverse resistance phenotypes to characterize resistance determinants, population structure and phylogenetic relationships, with long-read sequencing used for selected isolates to resolve plasmid architecture. Core-genome phylogenetic analyses were conducted for dominant sequence types (ST) to assess relatedness to international strains. Detailed genomic sequencing workflows and bioinformatic analyses are described in Supplementary Material 1 (available as Supplementary data at *JAC* Online).

### Statistical analyses

Quantitative resistance levels were analysed using linear mixed-effects models accounting for antimicrobial phenotype, year and hierarchical sampling structure. Temporal trends and phenotype-specific resistance patterns were assessed using separate models, with statistical significance set at *P* < 0.05. Full statistical methods and data visualization approaches are described in Supplementary Material 1.

## Results

### Resistance trends across herds

**Figure 2.**
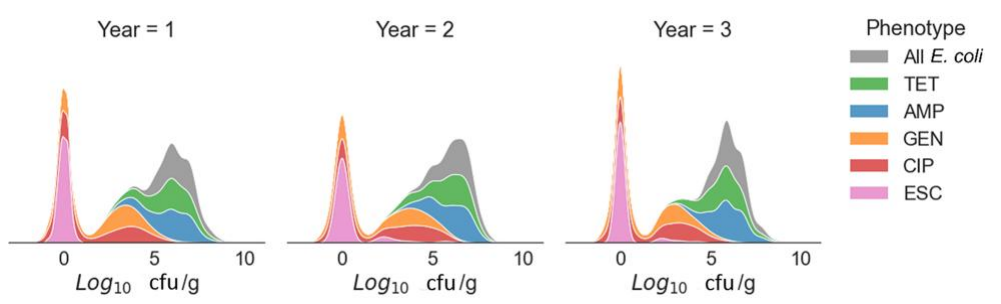
Probability distributions of log_10_ cfu/g counts of commensal *Escherichia coli* (including AMP, TET, GEN, CIP and ESC-resistant phenotypes) in pig faeces, estimated as kernel densities from 899 samples collected over 3 years from 10 herds.

*Escherichia coli* was detected in all 899 collected faecal samples except for two. Ampicillin, tetracycline, gentamicin and ciprofloxacin resistance were observed in all 10 herds, while only two herds were positive for ESC resistance (Figure 2). Resistance to ampicillin and tetracycline was detected in nearly all faecal samples, with prevalence rates of 99.8% and 99.0%, respectively. Median shedding levels for both antimicrobials remained high, consistently exceeding 5.8 log_10_ cfu/g across the 3 years. This high prevalence and density reflect widespread historic and contemporaneous use of these antimicrobial classes in Australian pig farming. In contrast, gentamicin resistance exhibited a significant decline over time, with shedding levels decreasing by −0.23 log_10_ cfu/g per year (*P* < 0.0001). This decline suggests reduced selective pressure or possible fitness costs associated with gentamicin resistance genes. Ciprofloxacin resistance was present at much lower levels (∼2.1 log_10_ cfu/g), with 58% of samples testing positive. Resistance levels for ciprofloxacin remained relatively stable throughout the study period. ESC resistance was rare initially but increased significantly over the final 2 years, with an annual increase of 0.16 log_10_ cfu/g (*P* = 0.015). This trend was confined to two herds, suggesting localized emergence rather than widespread dissemination. The maximum shedding levels of ESC-resistant *E. coli* were markedly lower than those observed for other resistance phenotypes, typically below 2 log_10_ cfu/g, indicating a low prevalence within the total *E. coli* population.

### Within-herd variability

**Figure 3.**
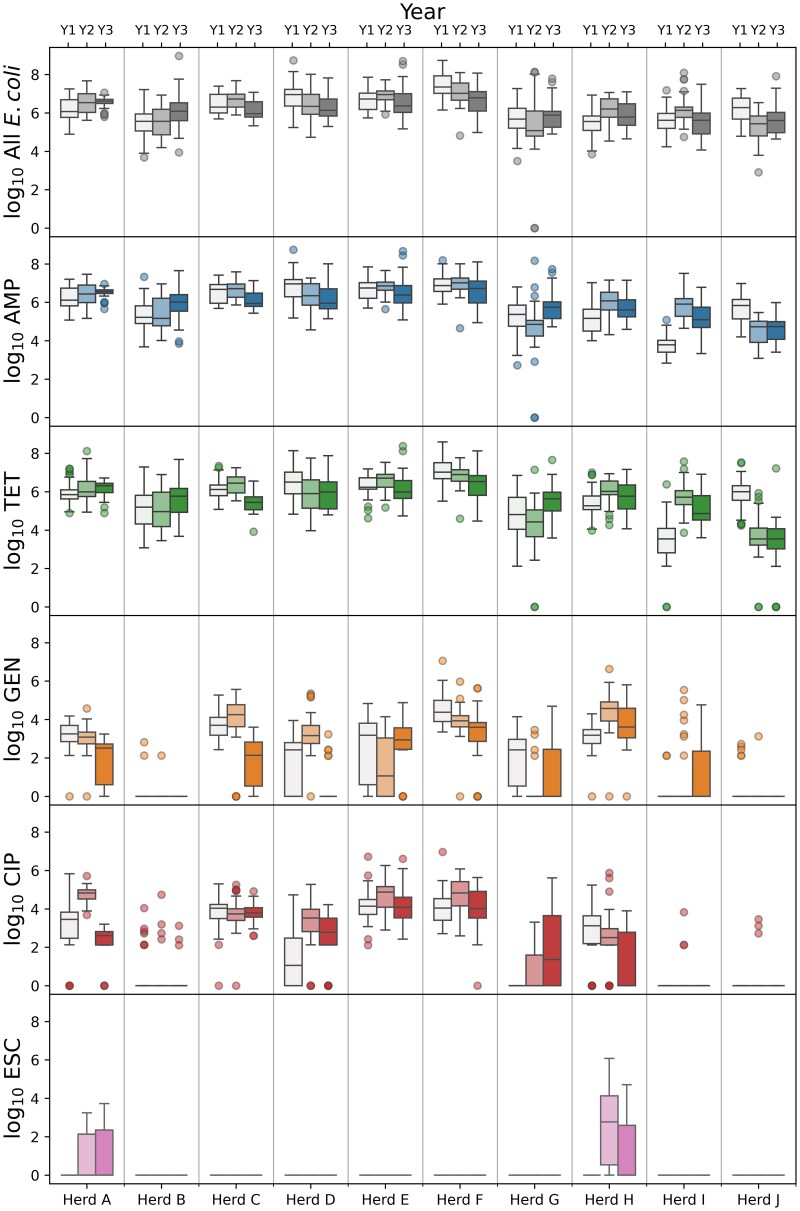
Breakdown of observed variation in the log_10_ cfu/g count of commensal *Escherichia coli* in pig faeces, one row for each phenotype: total *E. coli* (All *E. coli*), then resistant to ampicillin (AMP), tetracycline (TET), gentamicin (GEN), ciprofloxacin (CIP) and extended spectrum cephalosporin (ESC). Each column refers to one of 10 herds with three consecutive years of data within each herd. Individual box plots represent findings for thirty animals (except 29 samples for Herd F Year 1). Total number of samples and enumerations were 899 and 5394, respectively.

Resistance patterns varied substantially within and between herds (Figure 3). Ampicillin and tetracycline resistance were ubiquitous across all herds, with minimal variability in shedding levels. These phenotypes exhibited high interquartile ranges (5.5–6.2 log_10_ cfu/g), indicating consistently dense colonization by resistant *E. coli*. Gentamicin and ciprofloxacin resistance displayed greater variability. Some herds (e.g. Herd B and Herd I) showed very low levels or absence of resistance, while others exhibited higher prevalence rates. ESC resistance was uniquely confined to Herds D and E, where it emerged only during the second and third years. Within these herds, ESC resistance was detected in less than 10% of samples, but the shedding levels in positive samples were consistently low compared to other antimicrobials. Except where only a few isolates were detected, within-herd variation was consistently notable even for less prevalent resistance phenotypes such as ciprofloxacin and ESC resistance. Individual animals within the same herd exhibited shedding levels that varied by up to three log_10_ cfu/g, underscoring the importance of sampling multiple animals to accurately assess herd-level resistance.

### Isolate-level antimicrobial resistance

A total of 518 *E. coli* were isolated from fluoroquinolone screening plates (CIP) and were tested over 3 years using the RASP microdilution method.^6^ All isolates were confirmed resistant to ciprofloxacin based on CLSI clinical breakpoints. Most isolates also showed high resistance to ampicillin, chloramphenicol, streptomycin and tetracycline, with resistance to sulfamethoxazole/trimethoprim increasing to 100% over the study period (Table 1). Low resistance to florfenicol, gentamicin and apramycin was observed, while no resistance to cefoxitin, ceftiofur, ceftriaxone and colistin was detected. Nearly all isolates (516/518) were MDR, with the most common phenotype being resistance to six classes of antimicrobials. From the ESC screening plates (ESBL), 62 *E. coli* isolates from two farms were tested over 2 years. All isolates were confirmed ESC-resistant with MICs above CLSI clinical breakpoints for ceftiofur and ceftriaxone (Table 2). High resistance to ampicillin, sulfamethoxazole/trimethoprim and tetracycline was observed, while chloramphenicol resistance decreased significantly in the third year. Low resistance to streptomycin, ciprofloxacin, florfenicol and cefoxitin was also noted, with no resistance to apramycin, colistin and gentamicin. All 62 isolates were MDR, with the major phenotype in the second year being resistance to beta-lactams, third-generation cephalosporins, folate pathway inhibitors, phenicols and tetracyclines.

**Table 1.** Multidrug-resistance profiles of ciprofloxacin-resistant *Escherichia coli* isolates for all 3 years based on MIC estimation by microbroth dilution and application of clinical interpretive breakpoints

Multi-drug resistance profile	Antimicrobial classes *n*	First year *n* = 160		Second year *n* = 187		Third year *n* = 169	
		*n*	%	*n*	%	*n*	%
ami-bla-qui	3	48	30.0	60	32.1	0	0.0
bla-fpi-qui	3	0	0.0	0	0.0	1	0.6
bla-qui-tet	3	7	4.4	1	0.5	0	0.0
fpi-phe-qui	3	0	0.0	0	0.0	2	1.2
fpi-qui-tet	3	0	0.0	0	0.0	1	0.6
phe-qui-tet	3	1	0.6	3	1.6	0	0.0
ami-bla-c3g-qui	4	0	0.0	2	1.1	0	0.0
ami-bla-fpi-qui	4	0	0.0	0	0.0	29	17.0
ami-bla-phe-qui	4	0	0.0	1	0.5	0	0.0
ami-bla-qui-tet	4	1	0.6	1	0.5	0	0.0
bla-fpi-phe-qui	4	1	0.6	0	0.0	0	0.0
bla-fpi-qui-tet	4	0	0.0	0	0.0	9	5.3
bla-phe-qui-tet	4	11	6.9	4	2.1	0	0.0
fpi-phe-qui-tet	4	0	0.0	0	0.0	5	2.9
ami-bla-fpi-phe-qui	5	2	1.3	0	0.0	0	0.0
ami-bla-fpi-qui-tet	5	15	9.4	12	6.4	24	14.0
ami-bla-phe-qui-tet	5	0	0.0	1	0.5	0	0.0
bla-fpi-phe-qui-tet	5	23	14.4	14	7.5	52	30.4
ami-bla-fpi-phe-qui-tet	6	51	31.9	88	47.1	46	26.9

Isolates with resistance towards three or more antimicrobial classes are categorized as multidrug-resistant.

ami: aminoglycoside, bla: beta-lactam, c3g: third-generation cephalosporin, fpi: folate pathway inhibitor, phe: phenicol, qui: quinolone, tet: tetracycline.

**Table 2.** Multi-drug resistance profiles of ESC-resistant *Escherichia coli* isolates for Year 2 and Year 3 (no isolates were detected in Year 1) based on MIC estimation by microbroth dilution and application of clinical interpretive breakpoints

Multi-drug resistance profile	Antimicrobial classes *n*	Second year *n* = 38		Third year *n* = 24	
		*n*	%	*n*	%
bla-c3g-fpi-tet	4	4	10.5	8	33.3
ami-bla-c3g-fpi-tet	5	1	2.6	4	16.7
bla-c3g-fpi-phe-tet	5	21	55.3	6	25.0
bla-c3g-fpi-qui-tet	5	1	2.6	0	0.0
ami-bla-c2g-c3g-fpi-tet	6	0	0.0	1	4.2
ami-bla-c3g-fpi-phe-tet	6	10	26.3	4	16.7
bla-c2g-c3g-fpi-qui-tet	6	0	0.0	1	4.2
ami-bla-c3g-fpi-phe-qui-tet	7	1	2.6	0	0.0

Isolates with resistance towards three or more antimicrobial classes are categorized as multidrug-resistant.

ami: aminoglycoside, bla: beta-lactam, c2g: second-generation cephalosporin, c3g: third-generation cephalosporin, fpi: folate pathway inhibitor, phe: phenicol, qui: quinolone, tet: tetracycline.

### Dominant sequence types

Genome sequencing revealed that FQ-resistant isolates were dominated by two STs: ST744 (52.2% of isolates) and ST167 (41.2% of the isolates) (Table 3). Both lineages have been previously reported in humans, livestock and wildlife, reflecting their broad ecological adaptability and global dissemination. ST744 was the most prevalent lineage, accounting for the majority of isolates in the second (57.1%) and third years (54.2%). FQ resistance in these isolates was primarily driven by chromosomal point mutations in the quinolone resistance-determining regions of the *gyrA* and *parC* genes. Additionally, 12% of isolates harboured *qnrS1,* a plasmid-mediated FQ resistance gene.

**Table 3.** MLST of CIA-resistant *E. coli* isolates from Australian pigs

ST	Cip-Agar or cip-resistant *n* (%)				ST	ESC agar or ESBL-resistant *n* (%)		
	Total	Year 1	Year 2	Year 3		Total	Year 2	Year 3
744	71	22 (44.9)	36 (50.7)	13 (54.2)	1411	12	9 (60.0)	3 (60.0)
167	56	22 (44.9)	25 (44.6)	9 (37.5)	10	6	6 (40.0)	0 (0.0)
11613	2	0 (0.0)	2 (3.2)	0	1692	1	0	1 (20.0)
44	1	0 (0.0)	0 (0.0)	1 (4.2)	648	1	0	1 (20.0)
10	1	1 (2.0)	0 (0.0)	0				
11611	1	1 (2.0)	0 (0.0)	0				
11612	1	1 (2.0)	0 (0.0)	0				
34	1	1 (2.0)	0 (0.0)	0				
16348	1	0 (0.0)	0 (0.0)	1 (4.2)				
16349	1	1 (2.0)	0 (0.0)	0				

### Genetic context of extended-Spectrum cephalosporin resistance

ESC resistance was attributed to the *bla*_CTX-M-1_ gene, which was identified on IncI1 plasmids in all resistant isolates on heterogenous clones of *E. coli* (ST1141, ST10 and ST1629) (Table 3). Long-read sequencing confirmed that *bla*_CTX-M-1_ was plasmid-borne (pCTXM1-MU3), along with additional resistance genes, including *tet*(A) and *aadA5*, conferring resistance to tetracyclines and aminoglycosides, respectively. Plasmid analysis revealed a highly conserved structure, suggesting clonal dissemination of a single plasmid variant within the affected herds (Figure S1). A second ESBL gene, *bla*_CMY-2_, was identified with two non-contiguous copies in the chromosome of an isolate belonging to ST648.

### Genetic diversity

**Figure 4.**
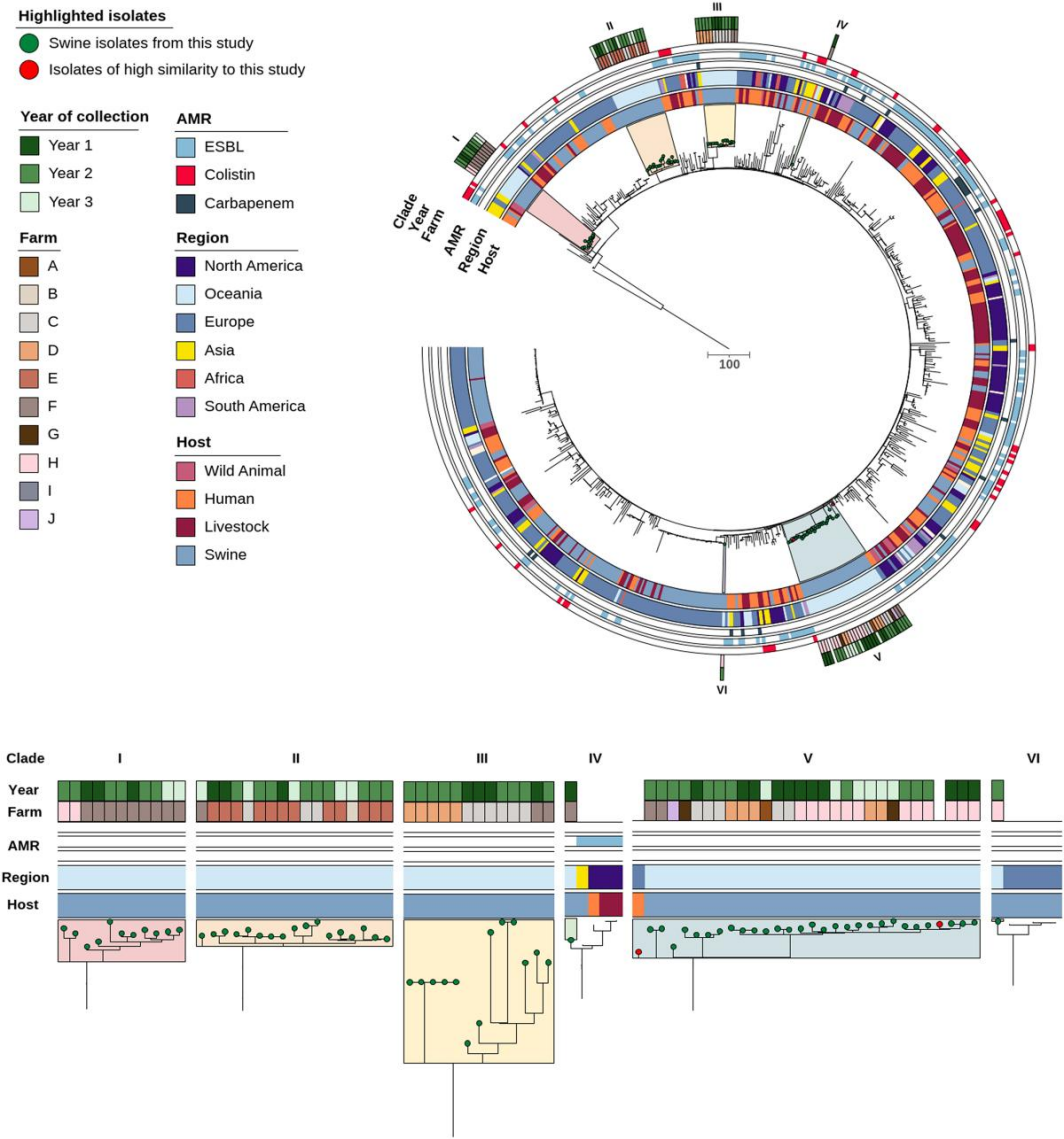
Phylogenetic comparison between ciprofloxacin-resistant *E. coli* strains identified as members of ST744 in this study and international ST744 strains obtained from EnteroBase based on core genome analysis. Strains involved in this study are shaded. The phylogeny was built on SNP calling in 67% (3.28 Mb) of the reference chromosome. Scalebar indicates SNPs.

**Figure 5.**
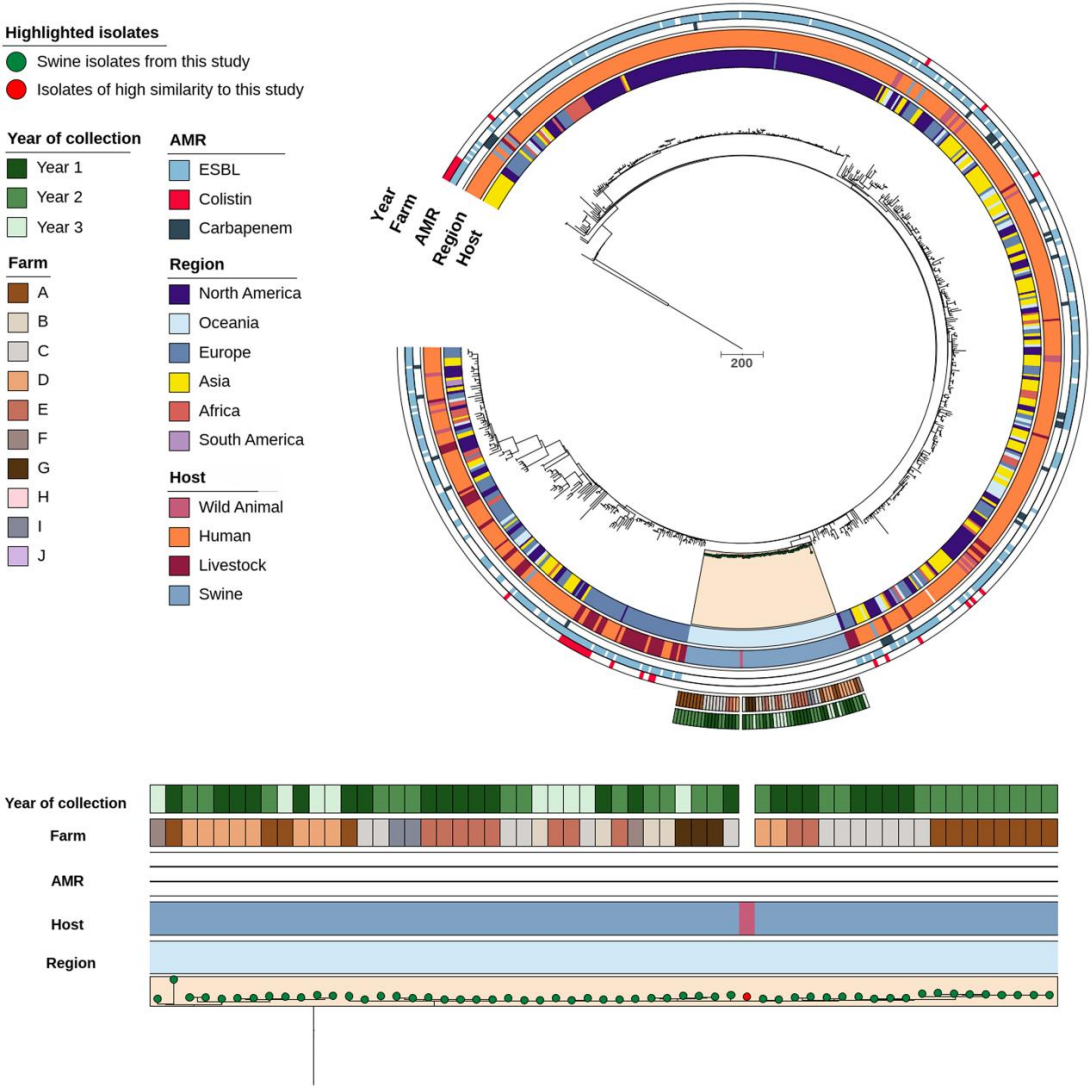
Phylogenetic analysis of Australian ciprofloxacin-resistant ST167 *E. coli* isolates and international ST167 isolates obtained from EnteroBase. Australian ST167 strains from this study were found clustered together (shaded area). The phylogeny was built on SNP calling in 58% (2.97 Mb) of the reference chromosome. Scalebar indicates SNPs.

In addition to ST744 and ST167, several other STs were identified among ciprofloxacin-resistant isolates, including ST10, ST44 and ST1629 (Table 3). These lineages were less frequent but exhibited resistance profiles consistent with those of dominant STs. Phylogenetic analysis revealed that Australian ST744 and ST167 isolates clustered closely with international isolates, suggesting multiple introduction events rather than local evolution (Figures 4 and 5). ST744 isolates formed six distinct phylogenetic clusters, with no evidence of clustering based on year or herd of origin. This suggests that multiple genetically distinct clones of ST744 were introduced into Australian pig herds over time. In contrast, ST167 isolates formed a single monophyletic clade, consistent with a single introduction event.

### Resistance dynamics over time

**Figure 6.**
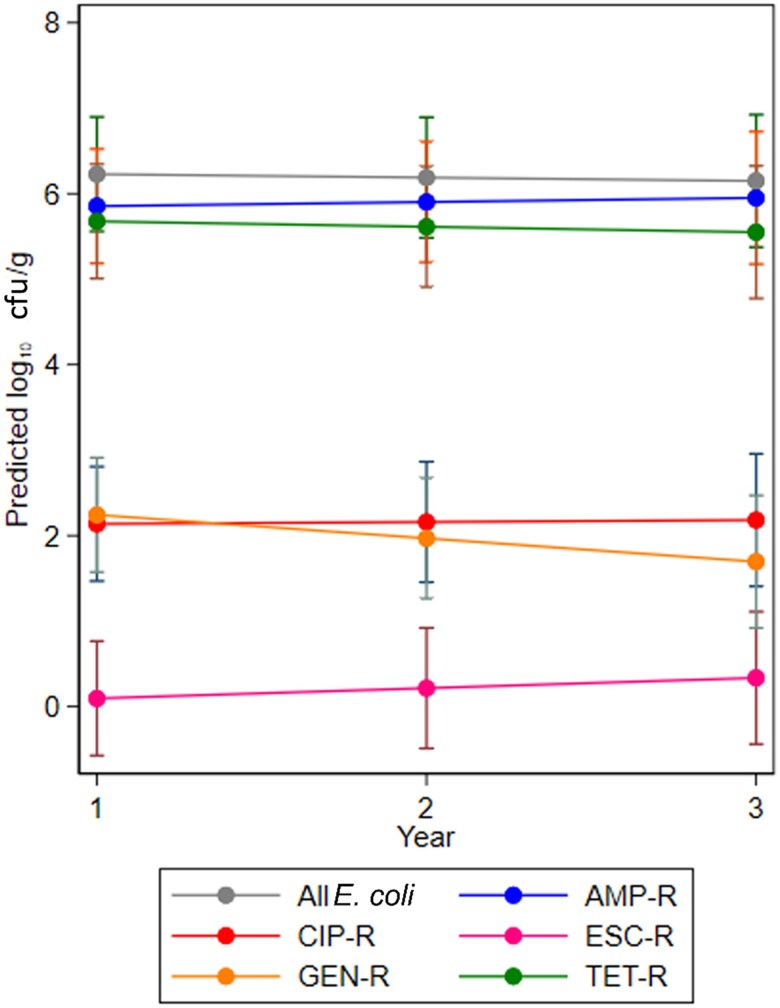
Mixed effect model predictions of the mean effect of resistance phenotype from the screening media plates and year of study on the log_10_ cfu/g count of *Escherichia coli* in pig faeces (total number of faecal samples = 5394 and total number of herds = 10). Phenotypes: Total *E. coli (All E. coli*), then resistant to ampicillin (AMP), tetracycline (TET), gentamicin (GEN), ciprofloxacin (CIP) and extended-spectrum cephalosporin (ESC).

Linear mixed-effect models quantified the temporal trends in resistance levels across all phenotypes (Figure 6). Ampicillin and tetracycline resistance showed no significant changes over the 3 years, maintaining consistently high levels. Gentamicin resistance, however, decreased significantly, with an estimated annual reduction of 0.23 log_10_ cfu/g (*P* < 0.0001). This trend for reversion to susceptibility aligns with reduced aminoglycoside use in Australian pig farming. ESC resistance demonstrated a contrasting pattern, with an annual increase of 0.16 log_10_ cfu/g (*P* = 0.015). This emergence was particularly evident in the second and third years, corresponding to the detection of *bla*_CTX-M-1_ plasmids in Herds D and E. The absence of ESC resistance in the first year suggests recent introduction and rapid dissemination within these herds.

### Global phylogenetic connections

Phylogenetic comparisons with international isolates provided insights into the potential origins of resistance in Australian pig herds. Australian ST744 isolates clustered with strains from humans, livestock and wildlife in Europe, Asia and North America (Figure 4). The global distribution of ST744 suggests that this lineage is well-adapted to multiple hosts and environments. ST167 isolates were closely related to strains from Europe and the Americas, indicating a possible introduction via humans or wildlife as there is regulatory and quarantine restriction on the importation of livestock and fresh meat products into Australia (Figure 5).

**Figure 7.**
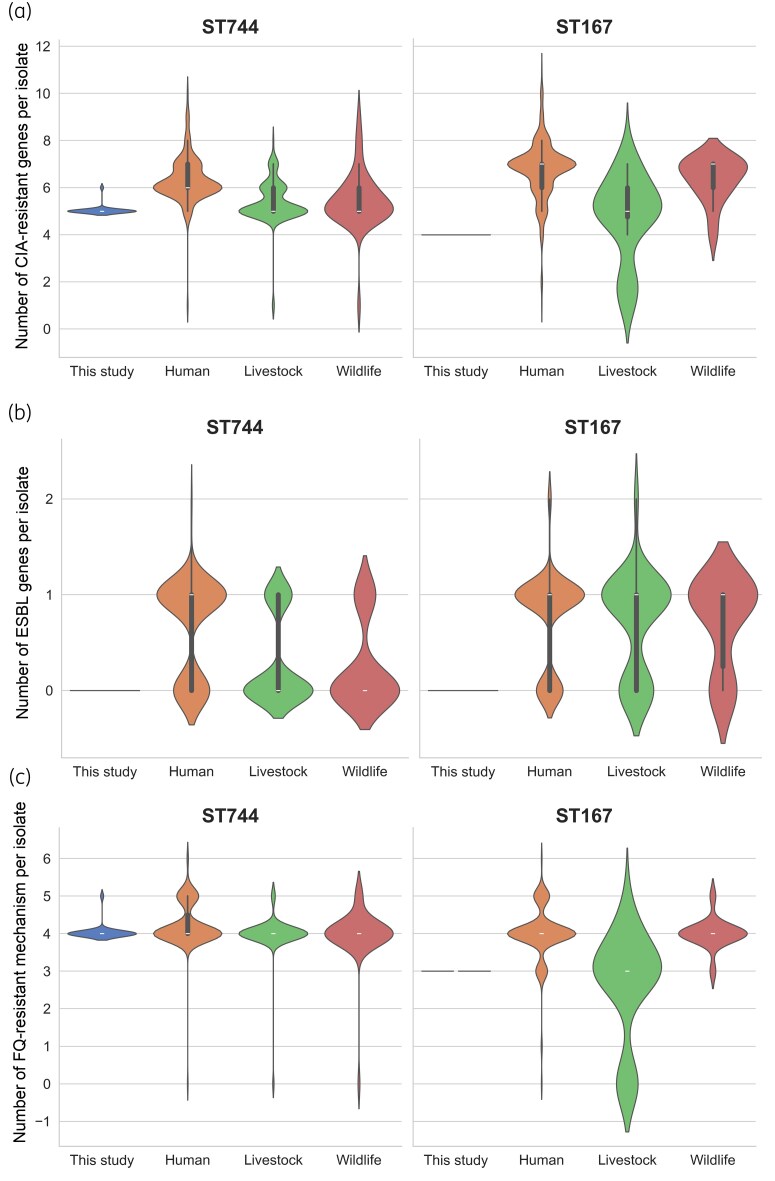
Comparison of WGS data for ST167 and ST744 *Escherichia coli* isolates from Australian pigs (*n* = 127) in this study and an international collection of isolates from humans (*n* = 623), livestock (*n* = 348) and wildlife (*n* = 56). Data are expressed as violin plots showing differences in the distribution of the number of antimicrobial resistance genes. (a) Number of CIA-resistant genes per isolate. (b) Number of Extended-spectrum β-lactamase (ESBL) genes per isolate. (c) Number of FQ-resistant mechanism per isolate.

Notable differences were observed in the carriage of total AMR genes and CIA-related resistance genes among Australian pig-derived ST744 and ST167 (this study) in comparison to humans, livestock and wildlife (Figure 7). Overall, Australian pig-derived ST744 and ST167 isolates carried a low number of AMR genes compared to humans, livestock and wildlife (Figure 7).

## Discussion

This study highlights the ecological complexity of AMR in livestock, focusing on *E. coli* from Australian pig herds. By integrating high-resolution phenotypic quantification with genomic analysis, this study generates operationally actionable information, enabling early detection of emerging resistance, herd-level stratification of AMR risk and targeted evaluation of antimicrobial use and stewardship interventions over time. We provide actionable insights into resistance trends and their implications for public health.

Ampicillin and tetracycline resistance, nearly universal across herds, reflects persistent selective pressures due to historical antimicrobial use.^7^ These findings emphasize the need for stewardship programmes to mitigate the entrenched presence of resistance in commensal bacteria. In contrast, the significant decline in gentamicin resistance demonstrates the potential for reducing resistance through decreased antimicrobial usage, reinforcing the importance of targeted interventions. Finally, the emergence of ESC resistance, confined to two herds, is of particular concern, due to its association with *bla*_CTX-M-1_ on IncI1 plasmids. These mobile genetic elements have significant dissemination potential, raising risks of horizontal gene transfer.^12^ Although ESC resistance was rare and localized, its increasing prevalence over time highlights the need for early detection and containment to prevent broader dissemination.

This study uniquely and conclusively demonstrated how the inclusion of validated enumeration assays based on agar dilution can enhance AMR surveillance by delivering a more detailed description of AMR (especially, in this case, FQ and ESC resistance with a low frequency) at the herd-level that would not be possible with established approaches to AMR surveillance based on a single isolate per herd.^13^ These are three examples why this model for monitoring resistance deserves broader application in the global livestock sector. The first is the ability to deliver robust evidence that a population of bacteria have reverted to a state of greater susceptibility to a specific antimicrobial, in this case gentamicin. The second is the use of RASP in early detection of emergence to a CIA (in this case ESC) to not only detect its overall presence in the entire population but also its occurrence in specific herds. Thirdly, despite the extensive ‘noise’ arising from within- and between-herd variation in amounts of tetracycline and ampicillin resistance, the technique of RASP combined with robust study design and analysis demonstrates a stable level of resistance for the period of the study. Importantly, these findings are actionable because they move AMR surveillance beyond binary presence–absence data. Quantitative enumeration enables early detection of low-prevalence resistance before fixation, allowing targeted, herd-level follow-up. For example, localization of ESC resistance to two herds supports focused investigation of antimicrobial use, biosecurity and introduction pathways, rather than sector-wide responses. Likewise, the observed decline in gentamicin resistance provides quantitative evidence of stewardship effectiveness, informing refinement of antimicrobial policies.

The ability to follow up isolates from solid and selective media provides the approach with special advantages since most of the ecologically important *E. coli* isolates in the population would otherwise be hidden within a vast myriad of benign isolates. Phenotypic and genotypic characterization of a selection of the CIA (ciprofloxacin and ESBL)-resistant isolates demonstrated the diversity within the populations. All except two CIA-resistant isolates selected had phenotypic resistance to three or more classes of antimicrobials, and all except one MDR phenotypes had resistance to beta-lactams (such as ampicillin) and/or tetracyclines, both of which are routinely used in the Australian pig industry. Here, ceftiofur (ESCs) are historically used off-label occasionally for treating respiratory infections thus explaining the presence of this form of resistance.^7^ It has been shown previously that ESBL-resistant isolates can be maintained in the commensal flora for at least 3 years after discontinuation of use.^14^ Considering that co-resistance to both tetracycline and ampicillin was a feature of all the ESBL-resistant isolates, it is possible that ESBL resistance will be maintained due to co-selection if the genes are clustered together or contained on the same plasmid, as is the case with the plasmid characterized in this study. When compared to a similar ESBL resistance plasmid isolated in previous studies the resistance determinant regions of both plasmids overlapped, however, the plasmid isolates in this study were larger in size than the previously isolated plasmid.^14^ The high prevalence of ampicillin- and tetracycline-resistant *E. coli* across Australian pig farms reflects the regular use of these antimicrobials.^7^ Although gentamicin is not approved for use in Australian food animals, the detection of gentamicin-resistant *E. coli* likely results from cross-resistance to other registered aminoglycosides such as neomycin, apramycin and spectinomycin.^15^ Notably, FQ-resistant *E. coli* in this study showed resistance to streptomycin but not apramycin or gentamicin, despite carrying aminoglycoside-resistance genes. Continued use of aminoglycosides in pigs could promote the acquisition of resistance to apramycin and gentamicin through cross-resistance mechanisms.

Whole-genome sequencing revealed ST744 and ST167 as dominant FQ-resistant lineages. These STs are globally significant, with links to isolates from humans, livestock and wildlife.^14,16–22^ The dual mechanisms of resistance—chromosomal mutations in *gyrA* and *parC* and the plasmid-mediated *qnrS1* gene—underscore the adaptability of these lineages in diverse environments. The identification of *bla*_CTX-M-1_ on plasmids also aligns with findings in other regions,^23–25^ suggesting potential introduction via humans, wildlife, imported breeding stock or environmental reservoirs. These findings underscore the role of complex resistance dynamics and global transmission pathways in shaping local resistance dynamics.

The presence of CIA-resistant *E. coli* in livestock poses direct and indirect risks to public health. Direct transmission via contaminated food products or environmental exposure can contribute to zoonotic infections with known pathogens such as *Salmonella enterica*. Indirect pathways, such as gene transfer to human pathogens,^3^ although poorly quantified, may act to amplify the challenge. The detection of globally significant lineages in Australian herds underscores the importance of coordinated One Health approaches to mitigate resistance spread. The low prevalence of ESC resistance in this study is encouraging but also a call for vigilance. The rapid emergence of resistance within isolated herds suggests that localized outbreaks can escalate quickly without targeted interventions. Moreover, interventions cannot be effectively targeted unless herd-level AMR status is known, which is a feature lacking in most if not all surveillance systems for AMR. Similarly, the persistence of ciprofloxacin-resistant *E. coli* despite the FQ class not being registered for use in Australian livestock highlights the role of co-selection by other antimicrobials or environmental pressures. This study demonstrates the feasibility and utility of integrating high-throughput robotic platforms and genomic tools for AMR surveillance. Robotic systems such as RASP enable robust sampling of host and microbial populations combined with standardized phenotypic quantification and opportunity for follow-through with genomic analyses to provide detailed insights into resistance mechanisms and transmission pathways. Together, these tools offer a sound scientific framework for monitoring emerging resistance and informing evidence-based policies. Automated high-throughput diagnostics is key to overcoming the longstanding barrier to progress linked to traditional culture-based methods which are labour-intensive and thereby impose restricted sample sizes and design constraints. High-throughput platforms allow for quantitative assessment of AMR phenotypes, capturing both common and low-prevalence resistance traits and changes in occurrence that are impossible to detect using traditional surveillance. The rapid processing when connected to automated reporting can deliver timely insights for decision-making in antimicrobial stewardship. Adopting similar surveillance systems globally could harmonize AMR monitoring efforts and improve comparability across regions. Most importantly, this enhanced surveillance can be used to define the AMR status of specific herds, which is the level at which key decisions about antimicrobial use are made. It provides the potential for motivating and empowering herd owners and veterinarians to align their own local actions with global stewardship goals.

### Conclusion

The findings from this study emphasize the importance of high-resolution AMR surveillance in understanding and managing resistance dynamics in livestock populations. By demonstrating the interconnectedness of AMR across regions and sectors, this research highlights the value of One Health approaches in mitigating risks. Future efforts should prioritize proactive monitoring, international collaboration and the integration of advanced surveillance technologies to address the growing challenge of AMR.

## Supplementary Material

dkag196_Supplementary_Data
