# Supplementary material for: High-resolution characterization of the temporal and spatial distribution of antimicrobial resistance in Escherichia coli from pigs
Source: J Antimicrob Chemother. 2026 Jun 19;81(7):dkag196. doi: 10.1093/jac/dkag196 (PMC13280645; doi:10.1093/jac/dkag196)
Supplement: dkag196_Supplementary_Data [file dkag196_supplementary_data.zip › Final_260131Supplementary_Material_1_reference.docx]

**Supplementary Material 1.**

**Genomic Analyses**

Whole-genome sequencing (WGS) was conducted on 151 isolates representing diverse resistance phenotypes. DNA was extracted using the ABI MagMax 96 Automated DNA Extraction System (Applied Biosystems, Thermo Fisher Scientific), which ensures high-quality nucleic acids suitable for sequencing. Libraries were prepared using the Nextera XT DNA Library Preparation Kit (Illumina, USA) and sequenced on an Illumina NextSeq 500 platform to generate paired-end reads of 150 base pairs. Raw sequencing reads were quality-checked using FastQC v0.12.1 ^1^ and trimmed with Trimmomatic v0.39 ^2^ to remove adapter sequences and low-quality bases. Reads were assembled *de novo* using the Unicycler pipeline v0.5.1 ^3^, and assemblies were annotated with Prokka ^4^. All genome assemblies were analysed for their sequence types (STs) with mlst v.2.16 ^5^. The genome assemblies were also screened using the abriTAMR pipeline v.1.0.14 for antimicrobial resistant determinants ^6^, virulence factors with the AMRFinderPlus v3.12.8 database abriTAMR ^7^ and serotypes were identified with ABRicate v.0.9.0 (https://github.com/tseemann/abricate) using the web-based analysis platform provided by the Center for Genomic Epidemiology (http://genomicepidemiology.org/services/).This analysis allowed for the identification of genetic lineages associated with specific resistance traits. Phylogenetic analyses were conducted on dominant STs (ST744 and ST167) to assess their relationships with international strains of ST744 and ST167 from EnteroBase. A core-genome SNP-based maximum likelihood phylogeny analysis was performed with the NASP pipeline v.1.1.0 ^8^ using as references for each ST the complete genomes ASM2269946v1 and ASM222402v1 representing ST167 and ST744, respectively. GATK v.4.2.2 was used for SNP calling and the recombination was removed using Gubbins (v.2.1)^9^. The phylogeny was created with IQ-TREE v.2.1.2 using ModelFinder and 100 bootstraps^10^.

Long-read sequencing using the Oxford Nanopore MinION platform was performed on four selected ESC-resistant isolates to resolve plasmid structures and confirm whether resistance genes were plasmid-borne or chromosomally integrated. Hybrid assemblies combining short- and long-read data were generated with Unicycler ^3^, enabling detailed (characterisation of plasmid architecture.

**Statistical Analyses µ**

Quantitative resistance levels, expressed as log_10_ cfu/g, were analyzed using linear mixed-effect models to evaluate the effects of antimicrobial phenotype and year. Herd and animal-level variability were included as random effects to account for hierarchical sampling. Model selection was guided by the Akaike Information Criterion (AIC) to ensure optimal fit.

Analysis of the design factors were conducted to compare resistance levels between years and identify significant temporal trends. Separate models were developed for each antimicrobial phenotype to evaluate distinct patterns of resistance dynamics.

Data visualization was performed using R (v4.1.2), Stata (v16.0, StataCorp) and Python (v3.11.7). Boxplots, probability density curves, and other graphical representations were used to illustrate variability within and between herds. Statistical significance was set at *p* < 0.05 for all analyses.

**References**

1. Brown J, Pirrung M, McCue LA. FQC Dashboard: integrates FastQC results into a web-based, interactive, and extensible FASTQ quality control tool. *Bioinformatics* 2017; **33**: 3137-9.

2. Truswell A, Lee ZZ, Stegger M *et al.* Augmented surveillance of antimicrobial resistance with high-throughput robotics detects transnational flow of fluoroquinolone-resistant *Escherichia coli* strain into poultry. *J Antimicrob Chemother* 2023; **78**: 2878-85.

3. Wick RR, Judd LM, Gorrie CL *et al.* Unicycler: Resolving bacterial genome assemblies from short and long sequencing reads. *PLoS Comput Biol* 2017; **13**: e1005595.

4. Seemann T. Prokka: rapid prokaryotic genome annotation. *Bioinformatics* 2014; **30**: 2068-9.

5. Jolley KA, Bray JE, Maiden MCJ. Open-access bacterial population genomics: BIGSdb software, the PubMLST.org website and their applications. *Wellcome Open Res* 2018; **3**: 124.

6. Sherry NL, Horan KA, Ballard SA *et al.* An ISO-certified genomics workflow for identification and surveillance of antimicrobial resistance. *Nat Commun* 2023; **14**: 60.

7. Feldgarden M, Brover V, Fedorov B *et al.* Curation of the AMRFinderPlus databases: applications, functionality and impact. *Microb Genom* 2022; **8**.

8. Sahl JW, Lemmer D, Travis J *et al.* NASP: an accurate, rapid method for the identification of SNPs in WGS datasets that supports flexible input and output formats. *Microb Genom* 2016; **2**: e000074.

9. Croucher NJ, Page AJ, Connor TR *et al.* Rapid phylogenetic analysis of large samples of recombinant bacterial whole genome sequences using Gubbins. *Nucleic Acids Res* 2015; **43**: e15.

10. Minh BQ, Schmidt HA, Chernomor O *et al.* IQ-TREE 2: New Models and Efficient Methods for Phylogenetic Inference in the Genomic Era. *Mol Biol Evol* 2020; **37**: 1530-4.

**Supplementary Figure Legends**


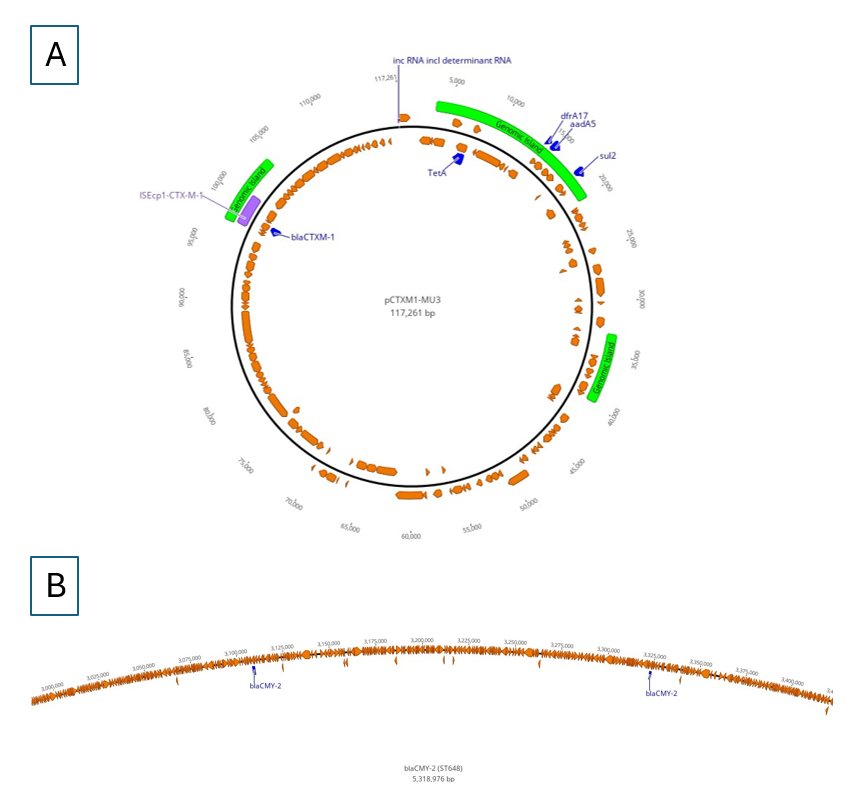


**Figure S1**. Genomic locations of ESBL genes identified. A) Circularized IncL1 plasmid pCTXM1-MU3 with three genomic islands identified two of which harbours antimicrobial resistance genes. B) Chromosomal location of two copies of *bla*_CMY-2_ in a single ST648 isolate 216,292 bp apart.
