# Supplementary figures and images for: High-resolution characterization of the temporal and spatial distribution of antimicrobial resistance in Escherichia coli from pigs
Source: J Antimicrob Chemother. 2026 Jun 19;81(7):dkag196. doi: 10.1093/jac/dkag196 (PMC13280645; doi:10.1093/jac/dkag196)

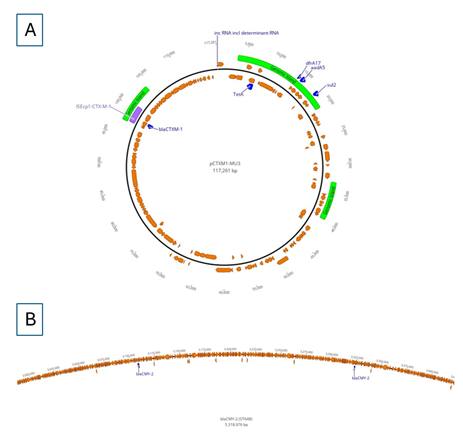

Supplement: dkag196_Supplementary_Data [file dkag196_supplementary_data.zip › Supplementary Figure S1_300.tif]
